# Active control of electroacoustic resonators in the audible regime: control strategies and airborne applications

**DOI:** 10.1038/s44384-025-00006-9

**Published:** 2025-04-07

**Authors:** Matthieu Malléjac, Maxime Volery, Hervé Lissek, Romain Fleury

**Affiliations:** 1 Laboratory of Wave Engineering, École Polytechnique Fédérale de Lausanne, Lausanne, Switzerland; 2 Univ. Bordeaux, CNRS, Bordeaux INP, I2M, UMR 5295, Talence, France

**Keywords:** Design, synthesis and processing, Mechanical engineering

## Abstract

Passive sound mitigation techniques have garnered attention whether for absorption, isolation, reverberation or new wave phenomena observation. In parallel, a wide range of research has been devoted to active control strategies, which complement passive techniques, particularly for low-frequency. We review the main control techniques related with airborne acoustic wave in the audible regime, emphasizing electrodynamic loudspeakers and piezo-diaphragms, and their applications. We conclude by discussing perspectives in this evolving field.

## Introduction

Designing lightweight structures to control sound has always been a challenge in airborne acoustics, particularly when it comes to low frequency absorption, diffraction pattern design, or achieving unconventional acoustic wave behaviors. To address these challenges, a variety of strategies have been developed, ranging from passive devices including locally resonant metamaterials, which operate without external power input, to active control systems that rely on external energy sources (e.g., electrical or mechanical) to enhance achievable performances. Although the first two will be briefly discussed below, this review will focus in particular on a subset of the latter, namely airborne active control strategies in the audible regime.

Sound absorption and insulation are core applications of acoustic treatments. Traditionally, porous materials such as mineral wool, foams, or fiber combinations have been used in front of a rigid backing. The interconnected network of pores (millimeters to micrometers) that make up these materials leads to visco-thermal losses in the boundary layers. To absorb effectively, the porous absorber must extend over a quarter of the acoustic wavelength, maximizing particle velocity at the interface between the treatment and the surrounding environment^1^. Due to this quarter-wavelength constraint, these treatments are suitable for addressing high audible frequency noise (typically above kilohertz), but are limited for lower frequency noise, which would require large dimensions for proper treatment. For instance, the material depth would usually need to extend over 1 m to treat a 340 Hz sound wave.

Several passive strategies have been developed to overcome these limitations. These include creating a gradient of material properties (porosity, tortuosity, etc.) along the porous frame^2,3^, filling resonators with optimized porous media^4^ or integrating resonators into the porous frame to improve low-frequency absorption properties^5,6^. Open lossy resonator structures have also proven effective in achieving near-perfect absorption and have been widely developed, from the well-known honeycomb lattice overlaid by a microperforated plate^7^ to more complex designs employing different types of resonators.

These locally resonant absorbing metamaterials rely on the critical coupling condition, which requires a perfect balance between the two counterparts of the *Q* factor. In other words, the system’s energy leakage part *Q*_*leak*_ and the inherent absorption part *Q*_*diss*_ of the resonator have to be equal, *Q*_*leak*_ = *Q*_*diss*_^8,9^. One of the main objectives of these structures is to be efficient at low frequencies while minimizing their volume. Coiled resonators^10,11^, Helmholtz resonators^12–14^, plates and membranes with added mass-platelets^15–19^, and bubble-screen^20^ are among the designs proposed for deep-subwavelength absorption either in reflection (one port)^21,22^ or in transmission (two-ports)^13,14,23^. One of the main limitations of such designs is the narrow frequency range associated with the resonant nature of the absorption which necessitates playing with collective resonances to broaden the working frequency band by coupling different resonators with different geometries^24–28^. Although the optimization of such structures enables deep-subwavelength broadband absorption, causality generally imposes limitations on the minimum achievable depth of passive devices^24,29,30^. It is important to note, however, that these limitations can be circumvented, e.g., by artificially adjusting the static bulk modulus (stiffness) of the medium^31,32^.

Passive devices can also control acoustic reflection, transmission, diffusion, and diffraction. While traditional treatments like Schroeder diffusers also face frequency-size limitations, the development of deep-subwavelength metamaterials and metasurfaces has enabled more precise control of acoustic waves. This includes Helmholtz resonator-based design that can time delay pulses^33^ or focus and steer the sound in a given direction with Helmholtz resonators^34^. Fine control of the diffusion and reverberation in a room can also be achieved using networks of Helmholtz resonators^35,36^ or bi-stable plates^37,38^. Additionally, non-mirror symmetric designs have also allowed an asymmetric control of acoustic waves giving rise to a new class of devices, the Willis materials, in which a coupling between potential and kinetic energy is observed^39–42^.

Despite the advancements in passive strategies, a major limitation is their lack of reconfigurability. Once designed, static structures can only be applied to specific scenarios and performance ranges. This has led to growing interest in active devices, which can be divided into two main categories: externally driven devices without or with acoustic energy injection.

Current research into time-varying materials mainly falls into the first category, where external mechanisms modify the medium’s properties without injecting energy into the acoustic field. These devices can switch between configurations for reconfigurable control of acoustic waves, such as transitioning from diffusive to absorptive behavior^43^, tuning absorption frequency ranges with moving walls^44^, or shaping wavefront. For the latter, a given pattern can be configured mechanically to form a targeted wavefront^34,37,45–47^ that can be used to focus in a given environment and even to create different communication channels without crosstalk in a disordered environment^34^. When modulated periodically^48^, unconventional propagation can occur such as non-reciprocal propagation^49,50^, amplification^51^, non-conventional circulation in multiport systems^52–54^, or even multi-harmonics diffraction control^55,56^.

The second category of active devices directly interacts and injects acoustic energy into the system, allowing real-time and reconfigurable control over sound propagation. Notable examples include phased arrays^57,58^ or active noise cancellation (ANC or anti-noise) devices that correctly remit the incident wave to perfectly cancel the incident sound^59–66^. The interested reader can refer to a recent Acoustics Today article on this topic^67^.

This review focuses on a sub-group of this second category, where an active control scheme is applied to directly modify the behavior of resonators, with particular emphasis on piezoelectric diaphragms and electrodynamic loudspeakers. The first section will therefore recall the model of these acoustic transducers, followed by sections devoted to the differentiation of the most commonly used control schemes, the different control bandwidths, and then various recent applications will be discussed.

## Electroacoustic transducer modeling

Electroacoustic transducers can generally be categorized into two main families based on their energy conversion mechanisms: those that utilize magnetic fields (e.g., electrodynamic, electromagnetic, and magnetostrictive transducers) and those that rely on electric fields (e.g., electrostatic and piezoelectric transducers). In this review, we focus exclusively on the modeling of electrodynamic and piezoelectric transducers, as they are the most widely studied and commonly employed actively controlled transducers in the literature.

### Electrodynamic loudspeaker modeling

**Fig. 1:**
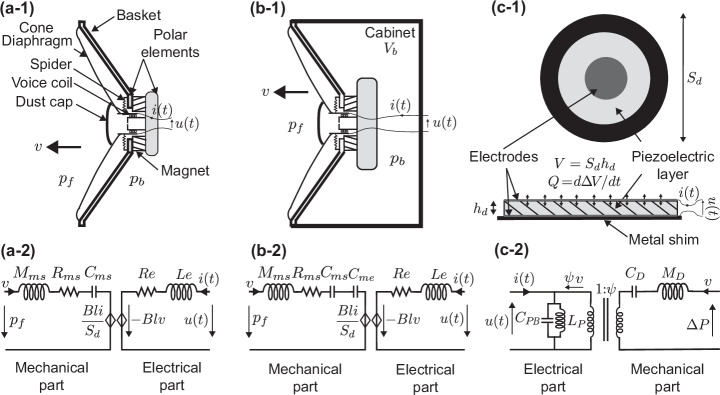
Schematic (.-1) & equivalent circuits (.-2) of loudspeaker and piezoelectric diaphragm. **a** In-tube electrodynamic loudspeaker. When an input current *i*(*t*) flows through the transducer voice coil, a Lorentz force is generated, forcing the diaphragm to move with a velocity $v=\dot{\xi }(t)$. The loudspeaker can be modeled by an equivalent electromechanical circuit **a-2** that involves the mass *M*_*ms*_, the resistance *R*_*ms*_, and the compliance *C*_*ms*_ of the membrane/diaphragm, as well as the electric resistance *R*_*e*_ and inductance *L*_*e*_. **b** closed-box loudspeaker. When the loudspeaker is embedded within a closed box/cabinet of volume *V*_*b*_, it reacts with added mechanical compliance ${C}_{me}={V}_{b}/({S}_{d}^{2}\rho {c}^{2})$. **c** Piezoelectric diaphragm. Under external voltage, the piezoelectric diaphragm reacts with a change in its volume Δ*V* characterized by *Q*, the volumetric change rate. As a result, a pressure drop across the diaphragm is generated. The equivalent circuit is composed of the blocked capacitance of the piezo-diaphragm *C*_*PB*_, and the mechanical compliance *C*_*D*_ and mass *M*_*D*_ of the diaphragm, linked through the electro-acoustic transduction coefficient *ψ*.

An electrodynamic loudspeaker is classically composed of a moving membrane with a diaphragm and a dome suspended to the basket by a spider and a suspension, acting as a mass-spring resonator as illustrated in Fig. 1a-1. When electrically actuated, the current *i*(*t*) flowing in the voice coil results in a Lorentz force, responsible for the motion *ξ*(*t*) of the membrane.

**Fig. 2:**
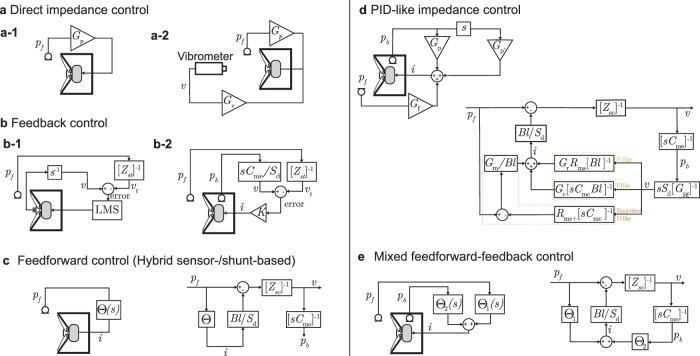
Schematic & block diagrams of the most used control strategies. *p* refers to the pressure (subscripts *f* and *b* refer to front and back respectively), *v* to the particle velocity, *G* and *K* to a constant proportional gain, and *Θ* to the control law (transfer function between *p* and *i*). **a** Direct impedance control with a single constant gain applied to the sensed pressure (**a-1**) and with constant gains applied to both the front pressure and the diaphragm velocity (**a-2**). **b** Feedback controller based on error minimization between the sensed velocity and the target velocity (obtained using the target impedance to be synthezised): P-V feedback using a microphone and a velocity sensor (accelerometer or laser vibrometer) **b-1** or P-only feedback where the velocity is estimated from the measured pressure gradient across the membrane **b-2**. **c** Hybrid sensor/shunt based control (feedforward type), requiring only the measurement of the front pressure *p*_*f*_. **d** PID (Proportional, Integral, Derivative) type control, which uses two pressure measurements to synthetically tune the mechanical properties of the loudspeaker independently with only three real constant gains. **e** Mixed feedforward and feedback control scheme that combines both strategies to improve the stability of the controller.

Additionally to the Lorentz force, three other forces act on the membrane, namely the front *p*_*f*_ and rear *p*_*b*_ acoustic pressures as well as the damping force ${R}_{ms}\dot{\xi }$, and the callback force exerted by the spring *ξ*/*C*_*ms*_, leading to the motion law of the membrane^68^

1 $${M}_{ms}\ddot{\xi }(t)={S}_{d}\left({p}_{f}(t)-{p}_{b}(t)\right)-{R}_{ms}\dot{\xi }(t)-\frac{\xi (t)}{{C}_{ms}}-Bli(t),$$

where the loudspeaker’s mechanical parameters (commonly named Thiele and Small parameters^69^) *M*_*ms*_, *R*_*ms*_, and *C*_*ms*_ are respectively the loudspeaker’s moving mass (kg), mechanical resistance (kg.s^−1^), and mechanical compliance (kg^−1^.s^2^), as shown in the equivalent circuit in Fig. 1a-2. *S*_*d*_ is the equivalent piston area, *Bl* is the force factor of the voice coil, and [^.^] and [^..^] refer to first and second order time derivatives respectively. The mechanical impedance of the loudspeaker therefore reads ${Z}_{ms}(\omega )={\rm{i}}\omega {M}_{ms}+{R}_{ms}+{\left[{\rm{i}}\omega {C}_{ms}\right]}^{-1}.$

The loudspeaker is commonly rigidly backed by a cabinet (closed-box) of volume *V*_*b*_, as illustrated in Fig. 1b-1. Below the first resonance of the box, the effect of the cabinet can be modeled as a spring accounting for the compression of the air (density *ρ* and sound speed *c*) contained in the box. The rear pressure can therefore be approximated as inversely proportional to the added mechanical compliance of the enclosure ${C}_{me}={V}_{b}/({S}_{d}^{2}\rho {c}^{2})$, namely ${p}_{b}(t)=\xi (t){\left[{S}_{d}{C}_{me}\right]}^{-1}$ and therefore allowing to eliminate the rear pressure in the motion equation

2 $${M}_{ms}\ddot{\xi }(t)+{R}_{ms}\dot{\xi }(t)+\frac{\xi (t)}{{C}_{mc}}={S}_{d}{p}_{f}(t)-Bli(t),$$

where ${\left[{C}_{mc}\right]}^{-1}={\left[{C}_{ms}\right]}^{-1}+{\left[{C}_{me}\right]}^{-1}$. The mechanical impedance of the closed-box loudspeaker therefore reads ${Z}_{mc}(\omega )={\rm{i}}\omega {M}_{ms}+{R}_{ms}+{\left[{\rm{i}}\omega {C}_{mc}\right]}^{-1}.$

Finally, the specific impedance of the in-tube or free field loudspeaker *Z*_*ss*_(*ω*) can be written as

3 $${Z}_{ss}(\omega )={\frac{\Delta p}{v}}_{| i = 0}=\frac{{Z}_{ms}}{{S}_{d}}={\rm{i}}\omega \frac{{M}_{ms}}{{S}_{d}}+\frac{{R}_{ms}}{{S}_{d}}+{\left[{\rm{i}}\omega {C}_{ms}{S}_{d}\right]}^{-1},$$

where Δ*p* = *p*_*f*_ − *p*_*b*_ is the pressure difference across the membrane, and the specific impedance of the closed-box loudspeaker *Z*_*sc*_(*ω*) as

4 $${Z}_{sc}(\omega )={\frac{{p}_{f}}{v}}_{| i = 0}=\frac{{Z}_{mc}}{{S}_{d}}={\rm{i}}\omega \frac{{M}_{ms}}{{S}_{d}}+\frac{{R}_{ms}}{{S}_{d}}+{\left[{\rm{i}}\omega {C}_{mc}{S}_{d}\right]}^{-1}.$$

### Piezoelectric diaphragm modeling

A piezoelectric diaphragm generally consists of a piezoelectric ceramic element sandwiched between two electrodes and bounded to a metal shim (e.g. nickel or brass) as shown in Fig. 1c-1. Once excited by an alternating voltage through the electrodes, the piezoelectric ceramic deforms, and alternatively stretches and retracts, leading to successive bending of the metal shim and the generation of a sound wave. In reverse, a pressure applied on the piezoelectric ceramic will generate a current. Such diaphragm has the advantage of being able to serve both of sensors and actuators.

The constitutive equations of the piezodiaphragm which relates the flow $Q={S}_{d}v(t)=\dot{\Delta V}$ (rate of the volume change) and the electric current *i*(*t*) to the pressure change Δ*p* and the applied tension *u*(*t*), read as

5 $$\left\{\begin{array}{c}Q\\ i\end{array}\right\}=\left[\begin{array}{cc}s{C}_{Dp}&sd{S}_{d}\\ sd{S}_{d}&s{C}_{P}\end{array}\right]\left\{\begin{array}{c}\Delta p\\ u\end{array}\right\}$$

where *C*_*P*_ = *S*_*d*_*ϵ*/*h*_*d*_ and *C*_*Dp*_ = *h*_*d*_*s*^*E*^*S*_*d*_ are the electrical and mechanical capacitance of the piezodiaphragm respectively (Farad and m^5^.N^−1^). *ϵ* is the stress-free permittivity of the piezoelectric ceramic (A^2^.s^4^.kg^−1^.m^−3^), *s*^*E*^ is the short-circuit compliance (m^2^/N), *d* is the piezo-strain coefficient (m/V), and *S*_*d*_ and *h*_*d*_ are the diaphragm’s cross-section area and thickness respectively^70,71^.

The pressure difference Δ*p* across the diaphragm

6 $$\Delta P=\psi u(t),$$

is related to the applied electric voltage *u*(*t*) through the electro-mechanic transformer ratio (also named effective piezoelectric coefficient) *ψ* = − *dS*_*d*_/*C*_*D*_ (s.A.m^−3^).

On the other hand, when the piezo-diaphragm is short-circuited, the pressure drop Δ*p* can be measured through the output charge of the diaphragm *q* = *dS*_*d*_Δ*p* or in other words, the measure of the current $i(t)=\dot{q}(t)$ flowing in the diaphragm gives a measure of the rate of pressure across the diaphragm

7 $$i(t)=d{S}_{d}\Delta \dot{p}.$$

The dynamic of the piezo-diaphragm is governed by^71–73^

8 $${M}_{D}\ddot{\xi }(t)+{Z}_{p}{\psi }^{2}{S}_{d}\dot{\xi }(t)+\frac{\xi (t)}{{C}_{D}}={S}_{d}\Delta p(t)-{Z}_{p}{S}_{d}\psi i(t).$$

and can be described by the equivalent electromechanical circuit shown in Fig. 1c-2. The mechanical impedance of the diaphragm is thus ${Z}_{mD}={\rm{i}}\omega {M}_{D}+{S}_{d}{Z}_{p}{\psi }^{2}+{\left[{\rm{i}}\omega {C}_{D}\right]}^{-1}$.

The mechanical part of the circuit comprises the mass of the piezoelectric diaphragm *M*_*D*_ = *ρ*_*d*_*h*_*d*_*S*_*d*_ (kg) and the compliance ${C}_{D}={C}_{DP}/{S}_{d}^{2}$ (s^2^.kg^−1^), while the electrical part consists of the impedance of the diaphragm ${Z}_{p}={\left[{\rm{i}}\omega {C}_{PB}+{[{\rm{i}}\omega {L}_{P}]}^{-1}\right]}^{-1}$ (Ohm), including the parallel arrangement of an inductance *L*_*p*_ (ensuring a density greater than that of the surrounding medium) and the blocked compliance of the diaphragm ${C}_{PB}={C}_{p}-{d}^{2}{S}_{d}^{2}/{C}_{D}$ (Farad).

As for the electrodynamic loudspeaker, one can define the specific impedance of such piezo-diaphragm

9 $${Z}_{sD}={\frac{\Delta p}{v}}_{| i = 0}=\frac{{Z}_{mD}}{{S}_{d}}={\rm{i}}\omega \frac{{M}_{D}}{{S}_{d}}+{\left[{\rm{i}}\omega {C}_{D}{S}_{d}\right]}^{-1}+{Z}_{p}{\psi }^{2}.$$

### Actively controlled transducers

The active control schemes applied to the transducer will aim at injecting a given current into it so that it responds according to a given control law. In other words, by injecting the proper current, we aim to modify the natural behavior of the transducer, and in particular, modify its specific impedance. The proper current is found in real-time from sensing one or several acoustic quantities nearby the loudspeaker membrane or piezodiaphragm, such as the acoustic pressures or its velocity, and applying various transformations. The most common control schemes are reviewed in the following section.

## Different control scheme

The goal of the section is to present the architectures of various control types. For sake of clarity, they are presented with loudspeakers, but similar control can be performed on piezoelectric diaphragms.

### Direct proportional impedance control

One of the earliest attempts to actively control the specific impedance of a closed-box loudspeaker was reported by Olson and May in 1953^74^. The proposed scheme depicted in Fig. 2a-1 consists of a direct control of the impedance by feeding the pressure *p*_*f*_ measured in front of the speaker directly back into the system after amplification by a constant gain factor *G*_*p*_. The impedance therefore reads as

10 $${Z}_{sa}=\left({Z}_{sc}+\frac{{(Bl)}^{2}}{{S}_{d}{Z}_{e}}\right){\left(1-\frac{{(Bl)}^{2}}{{S}_{d}{Z}_{e}}\frac{{G}_{p}}{Bl}\right)}^{-1},$$

where *Z*_*e*_(*ω*) = *R*_*e*_ + i*ωL*_*e*_ is the electric impedance, composed of the electric resistance *R*_*e*_ and inductance *L*_*e*_ of the voice-coil.

As the gain remains constant, this method does not modify the loudspeaker’s natural resonance frequency. However, it can alter the impedance magnitude and bandwidth. A key requirement for this control method is an accurate estimation of the loudspeaker’s parameters, which are typically calibrated beforehand through short- and open-circuit reflection measurements.

An improved version of the direct control^75^ can incorporate an additional constant gain on the velocity *G*_*v*_. This version requires direct measurement of the diaphragm velocity, which can be achieved using a laser-Doppler vibrometer (see Fig. 2a-2) or an accelerometer for instance. The controlled impedance is then modified as

11 $${Z}_{sa}=\left({Z}_{sc}+\frac{{(Bl)}^{2}}{{S}_{d}{Z}_{e}}\left(1+\frac{{G}_{v}}{Bl}\right)\right){\left(1-\frac{{(Bl)}^{2}}{{S}_{d}{Z}_{e}}\frac{{G}_{p}}{Bl}\right)}^{-1}.$$

Alternatively, other methods have been proposed to estimate the diaphragm velocity without expensive equipment. For instance, a second coil can be used to sense the induced voltage, which is proportional to the diaphragm’s velocity^76^.

As its name suggests, a direct proportional controller relates the sensor measurement directly to the actuator response. It is therefore simple to implement, well suited to real-time applications in simple scenarios involving systems with predictable dynamics. However, it struggles with complex systems, steady-state errors, and is sensitive to sensor noise. It also does not account for system’s saturation and can become inefficient or unstable with improper tuning or in the presence of non-linear dynamics.

### P-V feedback control

Another straightforward control method consists of applying a feedback loop to the loudspeaker. Typically, this approach requires measuring the pressure near the diaphragm and the diaphragm’s velocity *v*. Knowing the pressure, a target impedance can be synthesized by adequately choosing the diaphragm velocity. The feedback loop is then implemented by minimizing the error signal, which is the difference between the target and measured velocities. Various methods can be used to measure the diaphragm velocity, as mentioned earlier. These include a vibrometer^75^, a secondary coil^77^, or an accelerometer positioned directly on the diaphragm^78^ (see Fig. 2b-1).

The feedback control scheme theoretically relaxes the need for accurate transducer modeling. However, in practice for industrial applications, accurate characterization of both the transducer and sensors remains essential to achieve precise control in arbitrary sound environments. Moreover, as it is based on an error signal minimization, it is subjected to unavoidable discrepancies and potential stability issues. These challenges can be addressed through the use of compensators and adaptive filters, such as x Least Mean Square algorithm^79^. The significant drawback of the pressure-velocity feedback controller is thus the complexity of implementation, as it requires additional bulky velocity sensors and the implementation of adaptive filter and regulation techniques to stabilize the control.

### P-only feedback contoller

Accurate modeling of the actuator can in some circumstances not be possible (characterization not possible, abnormal dynamics, or modified actuator). Although P-V feedback strategies are suitable for such situations, the cost or high intrusiveness of the velocity sensors makes it necessary to develop robust P-only feedback “model-less” control schemes.

Initial attempts to address this challenge were made by Guicking et al.^80^ and Orduna-Bustamante et al.^81^, who positioned two microphones upstream of the diaphragm to estimate the acoustic impedance in real-time. The measurements were then fed into a static and adaptive controller respectively, to minimize an error signal between the measured and target impedance. These early works demonstrated high controllability of the synthesized impedance, particularly in the sub-kHz range. The use of the filtered X-LMS algorithm makes it possible to treat both transient and random noise in addition to periodic acoustic signals^81^.

Another strategy involves estimating the diaphragm’s velocity from pressure measurements using two microphones: one positioned in front of the diaphragm and the other inside the cavity, as shown in Fig. 2b-2. This approach draws inspiration from previous work by Meynial et al., who used a Wheatstone bridge to estimate the velocity by knowing the finely tuned resistance composing the bridge^82^, and from Darlington, who estimated the diaphragm’s velocity using the integrated acceleration signals^83^. However, the use of bulky accelerometers is not suitable for small loudspeakers, as the accelerometer mass can significantly impact the transducer’s dynamics. In the implementation proposed by Volery and Lissek^84^, the velocity of the diaphragm is estimated directly from the measured total pressure inside the cavity *p*_*b*_, which is proportional to the diaphragm displacement

12 $${p}_{b}(t)=\xi (t){[{S}_{d}{C}_{{\rm{me}}}]}^{-1},$$

where *ξ*(*t*) is the diaphragm displacement and *C*_me_ is the acoustic compliance of the enclosure, which can be easily estimated.

The diaphragm velocity $\tilde{v}$ is then estimated with a single microphone, by numerically differentiating the rear pressure and dividing it by the enclosure compliance. This estimated velocity is compared with the targeted velocity, obtained by dividing the front pressure, *p*_*f*_, by the target-specific impedance, *Z*_*st*_. The current to be injected into the voice coil to attain this target is then determined from the velocity difference (error signal) multiplied by a proportional controller with gain *GS*_*d*_/*Bl*

13 $$i(s)=G\frac{{S}_{d}}{Bl}\left(s{C}_{{\rm{me}}}{p}_{b}(s)-\frac{{p}_{f}(s)}{{Z}_{{\rm{st}}(s)}}\right).$$

It is worth noting that although the equivalent cross-section area, *S*_*d*_, and the Force factor, *Bl*, appear in the control law, their impact is superficial since they only act as a scaling factors for *G*.

The impedance achieved with this control scheme is given by

14 $${Z}_{sa}={Z}_{st}\frac{{Z}_{sc}+G}{{Z}_{st}+G}.$$

indicating that the specific impedance can never be exactly reached, as *Z*_*sa*_ = *Z*_*st*_ can only be achieved with infinite gain. The advantage of this controller is that it guarantees passivity for real target impedance since the loudspeaker remains in that case a single degree of freedom resonator. In contrast, if the target contains a reactive part, passivity is no longer guaranteed. This controller, although simply based on P-only feedback control without any additional compensator or adaptive filter, is perfectly suited and robust for the synthesis of reactive impedance, especially since it does not depend on the exact knowledge of the transducer dynamics nor on the time delay of the controller. It can therefore outperform feedforward controllers in certain cases where both the inaccuracy of the modeling and the delay of the controller can be detrimental.

### Hybrid Sensor-/Shunt-Based (feedforward) impedance control

Rather than using the loudspeaker as a secondary source to achieve the desired impedance at a specific position through error minimization, control strategies can be employed to directly synthesize a given specific impedance.

In addition to active control, passive shunting has also demonstrated its capability to modify the dynamics of a transducer. Specific impedance can be synthesized either analogically or digitally by shunting the transducer with electrical loads (such as positive or negative resistance, inductance)^75,85–88^, or through mechanical and electromechanical loads^77,89^. However, these passive shunting methods can be difficult to fine-tune and are generally not reconfigurable, as they lack field-programmable flexibility. Moreover, they do not allow for a reduction in actuator damping, except through negative resistance circuits, which could negatively impact control stability.

To overcome these limitations, a new class of feedforward active control schemes has been developed by merging the shunt-based method with direct pressure or velocity sensing, giving rise to the hybrid sensor/shunt-based impedance control^90^ as shown in Fig. 2c. Instead of controlling the loudspeaker with voltage, the control is performed directly on the current flowing through the actuator voice coil, which is proportional to the driving force on the diaphragm, so that electrical impedance can be bypassed. A voltage-controlled current source can be used for this purpose, e.g. using a Howland current pump^91^.

The control strategy involves designing an appropriate transfer function *Θ*(*s*) in the Laplace domain, which relates the total pressure in front of the transducer *P*_*f*_(*s*), to the injected electrical current *I*(*s*), in such a way that the system responds with a given specific target impedance *Z*_*st*_(*s*)

15 $$\Theta (s)=\frac{I(s)}{{P}_{f}(s)}=\frac{{S}_{d}}{Bl}\left(1-\frac{{Z}_{sc}(s)}{{Z}_{st}(s)}\right),$$

where *s* is the Laplace variable (*s* = i*ω*, with *ω* real if a Fourier transform substitutes the Laplace transform).

The target-specific impedance is typically defined as a modified spring-mass-damper system, which looks like the passive membrane impedance, but with added constant tuning parameters *μ*_*m*_, *μ*_*r*_, and *μ*_*c*_ to modify the specific mass, resistance, and compliance, respectively

16 $${Z}_{st}(s)=s{\mu }_{m}\frac{{M}_{ms}}{{S}_{d}}+{\mu }_{r}\frac{{R}_{ms}}{{S}_{d}}+{\mu }_{c}{\left[s{C}_{mc}{S}_{d}\right]}^{-1},$$

and the controller’s transfer function can therefore be written as follows

17 $$\Theta (s)=\frac{Sd}{Bl}\frac{s({\mu }_{m}-1)\frac{{M}_{ms}}{{S}_{d}}+({\mu }_{r}-1)\frac{{R}_{ms}}{{S}_{d}}+({\mu }_{c}-1){\left[s{C}_{mc}{S}_{d}\right]}^{-1}}{s{\mu }_{m}\frac{{M}_{ms}}{{S}_{d}}+{\mu }_{r}\frac{{R}_{ms}}{{S}_{d}}+{\mu }_{c}{\left[s{C}_{mc}{S}_{d}\right]}^{-1}}.$$

Typically, the tuning parameters are chosen as *μ*_*m*_ = *M*_*mt*_/*M*_*ms*_, *μ*_*c*_ = *C*_*mc*_/*C*_*st*_, and *μ*_*r*_ = *R*_*mt*_/*R*_*ms*_ to synthesize a target impedance ${Z}_{st}=s{M}_{mt}/{S}_{d}+{R}_{mt}/{S}_{d}+{\left[s{S}_{d}{C}_{mt}\right]}^{-1}$.

Although this control scheme does not require the knowledge of the speaker’s electrical impedance, the transfer function relies on *Z*_*sc*_, the specific impedance of the closed-box loudspeaker, and therefore requires an accurate characterization of the latter (i.e., *M*_*ms*_, *R*_*ms*_, *C*_*ms*_, *Bl*, and *S*_*d*_). Experiments have shown the ability of this feedforward controller to modify a given passive impedance in the deep subwavelength regime. Typically, the tuning parameters can be used to modify the impedance with up to a factor 10 in mass, compliance, and resistance, when the dynamics of the loudspeaker is well characterized. In practice, this method is ultimately limited by stability, that is sensitive to both the accuracy of the loudspeaker dynamics characterization and the Input-Output (I/O) latency of the digital controller^92^. De Bono et al. explored the interplay between this time-delay and the loss of passivity that leads to instabilities in the control. Because of the time-delay, the real transfer function achieved by the loudspeaker is different from the targeted one due to an additional phase-shift that increases with frequency, causing the loss of passivity. The higher the time-delay, the lowest the frequency at which the electroacoustic absorber loses its passivity and the higher the negative drop of the absorption coefficient.

### PID-like feedback controller

With a proportional controller, the loudspeaker’s dynamics are altered as a whole, since only a proportional gain is applied. To achieve more refined control, a more advanced strategy can be employed to independently control each of the actuator’s mechanical parameters, i.e. the moving mass, resistance, and compliance. One such approach is to implement a PID-like feedback control^93^, in which three different paths are used to fine-tune the different parameters related to the membrane’s velocity, i.e., *R*_*ms*_ which is proportional, ${[{C}_{ms}]}^{-1}$ that is integral, and *M*_*ms*_ that is differential. Inspired by this three branches control, an equivalent PID-like feedforward controller shown in Fig. 2d has been proposed^94,95^. The key idea is to avoid using an error signal and instead to synthesize the impedance more accurately by applying independent proportional, derivative, and integral gains to the pressures upstream of the diaphragm *p*_*f*_ and in the cabinet *p*_*b*_.

With such a controller, the output current reads as

18 $$i(s)={G}_{f}{p}_{f}(s)+{G}_{p}{p}_{b}+{G}_{p}^{{\prime} }s{p}_{b}.$$

The controller implies three different gains *G*_*f*_, *G*_*p*_, and ${G}_{p}^{{\prime} }$ in its simplest form leading to the specific impedance

19 $${Z}_{sa}=\frac{{Z}_{sc}+Bl({G}_{p}+s{G}_{p}^{{\prime} })/(s{C}_{me}{S}_{d}^{2})}{1-Bl{G}_{f}/{S}_{d}}.$$

To synthesize a target mass *M*_*mt*_, resistance *R*_*mt*_, and compliance *C*_*mt*_, the gains have to take the following expressions

20 $${G}_{f}=\frac{{S}_{d}}{Bl}\left(1-\frac{{M}_{ms}}{{M}_{mt}}\right),$$

21 $${G}_{p}=\frac{{S}_{d}}{Bl}\frac{{C}_{me}}{{C}_{mt}}\left(\frac{{M}_{ms}}{{M}_{st}}-\frac{{C}_{mt}}{{C}_{mc}}\right),$$

22 $${G}_{p}^{{\prime} }=\frac{{S}_{d}}{Bl}{C}_{me}{R}_{mt}\left(\frac{{M}_{ms}}{{M}_{st}}-\frac{{R}_{ms}}{{R}_{mt}}\right).$$

This controller has the great advantage that it also works with inaccurate modeling of the loudspeaker, as the combination of the three real gains always synthesizes a single degree of freedom passive absorber. It can also be easily implemented purely analog with basic analog components and offers an inexpensive option for impedance synthesis, although it is less straightforward to be tuned once set.

The block diagram in Fig. 2f shows a practical realization of such PID-like controller. Guo et al. defined three gains *G*_*m*_, *G*_*r*_, and *G*_*c*_ that are related to *G*_*f*_, *G*_*p*_, and ${G}_{p}^{{\prime} }$ through the following relations

23 $${G}_{f}=\frac{{G}_{m}{S}_{d}}{Bl},$$

24 $${G}_{p}=\frac{{C}_{me}}{SdBl}\left(\frac{{G}_{c}}{{C}_{me}}-\frac{{G}_{m}}{{C}_{mc}}\right),$$

25 $${G}_{p}^{{\prime} }=\frac{{R}_{ms}}{Bl}\frac{{C}_{me}}{{S}_{d}}\left({G}_{r}-{G}_{m}\right).$$

The achieved impedance is therefore

26 $${Z}_{sa}(s)={\mu }_{m}\frac{s{M}_{ms}}{{S}_{d}}+{\mu }_{r}\frac{{R}_{ms}}{{S}_{d}}+{\mu }_{c}{\left[s{C}_{mc}{S}_{d}\right]}^{-1},$$

where the classical control parameters read as ${\mu }_{m}=1{\left[1-{G}_{m}\right]}^{-1}$, ${\mu }_{r}=1+{G}_{r}{\left[1-{G}_{m}\right]}^{-1}$, and ${\mu }_{c}=1+{G}_{c}{\left[1-{G}_{m}\right]}^{-1}$. This controller has proven to be very robust against modeling inaccuracies thanks to the three control branches, as well as to efficiently compensate for the detrimental time delay. Reconfigurable absorption in the deep subwavelength region has been reported in terms of frequency and bandwidth in the range of 50–500 Hz. While the mass and compliance control parameters were used to select the absorption frequency (*μ*_*m*_ and *μ*_*c*_, which vary between 0.4 and 2), the resistance control parameter was used to adjust the synthetic resistance to achieve impedance matching (*μ*_*r*_ ∈ [1 − 4.5]). The authors have shown that their PID-like controller can outperform the feedforward controller, especially in the frequency range close to resonance, which is more sensitive to the adverse effects mentioned above.

The main advantage of using three independent controllers lies in their ability to provide flexibility in impedance synthesis by decoupling the individual dynamic parameters, while offering improved and robust stability. However, this approach is more complex to tune, prone to sensor noise, and can struggle with controlling nonlinear or dynamic systems.

### Mixed feedback-feedforward control

All the control strategies presented in the previous sections suffer either from a dependency on the accuracy of the actuator modeling and characterization or from an incapacity to perfectly synthesize the target impedance with feedback controls, as well as passivity loss. Volery et al. proposed to combine the advantages of both feedforward and feedback controllers for improved robustness^95,96^. The control scheme illustrated in Fig. 2e, inspired by the model-less feedback controller, uses two microphones, both related to a separate linear time-invariant control law *Θ*_1_(*s*) and *Θ*_2_(*s*) for the feedforward and feedback paths respectively

27 $$i(s)={\Theta }_{1}(s){p}_{f}(s)+{\Theta }_{2}(s){p}_{b}(s),$$

with

28 $${\Theta }_{1}(s)=\frac{{S}_{d}}{Bl}\left(1-\frac{{Z}_{sc}(s)+G(s)}{{Z}_{st}}\right),$$

and

29 $${\Theta }_{2}(s)=s\frac{{C}_{me}{S}_{d}^{2}G(s)}{Bl}.$$

Although the gain *G* can be chosen arbitrarily, to avoid any divergence and instability at high frequency, *G* can in particular take the form of a low pass filter of cut-off frequency *ω*_*g*_ and gain *k*_*g*_ > = 0,

30 $$G(s)=\rho c{k}_{g}\frac{{\omega }_{g}}{s+{\omega }_{g}},$$

to shortcut the feedback controller above the first cut-off frequency of the enclosure *ω*_*c*_ (i.e., *ω*_*g*_ < = *ω*_*c*_), above which the relation between rear pressure *p*_*b*_ and the membrane’s displacement *ξ*, is no longer valid.

By integrating both feedback and feedforward strategies, these controllers harness the strengths of both approaches, offering improved performance and robustness. However, they also come with greater complexity and increased computational requirements.

### Summary

Selecting the most appropriate active control strategy depends on the specific application being addressed, as it depends on factors such as sensor type, intrusiveness, and the inherent practical limitations and stability of each approach. In order to summarize this section, we have compiled in Table 1 the main advantages and disadvantages of the different control schemes presented above.

**Table 1:** Advantages and drawbacks of the different control strategies

Control strategies	Sensor #	Advantages	Drawbacks
Direct control	1	- Non intrusive, easy to implement, stable - Low requirement on the transducer knowledge	- Very limited control (single proportional gain)
P-V direct control	2	- Low requirement on the transducer knowledge	- Limited control (two proportional gains) - Intrusive (accelerometer) or expensive (laser vibrometer)
P-V feedback	2	- Error minimization (limited sensitivity to transducer modeling) - Wide choice of adaptive filtering techniques - Easy to implement	- Direct sensing of the velocity : intrusive or costly - Target impedance can only be approached (minimization) - Stability issues & sensitivity to disturbances
P-only feedback	2	- Error minimization (limited sensitivity to transducer modeling) - Wide choice of adaptative filtering techniques - Easy to implement Non intrusive	- Target impedance can only be approached (minimization) - Stability issues & sensitivity to disturbances
PID-like	2	- Impedance synthesis with only constant gains - Intuitive and easy to implement even analogically - Resilience to model inaccuracy	- Approximate in the high-frequency range (cabinet modeling inaccurate)
Feedforward	1	- Impedance synthesis (transfer function), high flexibility - Non intrusive -	- Sensitive to I/O latency - Very sensitive to modeling errors or system changes
Mixed feedforward	2	- Impedance synthesis (transfer function) - Combines the strengths of both feedback and feedforward techniques - Non intrusive - Improved stability - Enhanced resilience to I/O latency & modeling inacurracy	- Increased system complexity - Two transfer functions needed - Manual tuning of the sweet spot balance between feedforward and feedback

## Application of active control on single loudspeaker and liners

Having outlined the main active control strategies, this review will now focus on their applications. In this first section, we will explore applications related to modifying the properties of resonators, including sound absorption and room acoustic control.

### Synthetic modification of the resonance properties and the impedance

#### Broadband control: transfer function synthesis

As demonstrated in “Different control scheme”, most control laws operate over a broad frequency range, either through a direct proportional gain that modifies the entire specific impedance or through a carefully designed transfer function. This transfer function relates the measured total pressure in front of the membrane to the injected current, using the parameter *s* (or equivalently i*ω*), enabling effective control across a wide frequency spectrum.

In the latter approach, a specific impedance can be synthesized by appropriately selecting control parameters in the transfer function. This allows for tailoring the target resonance frequency *f*_*t*_ and the resonator bandwidth *Q*_*t*_ from their natural parameters (${f}_{mc}=2\pi {\left[{M}_{ms}{C}_{mc}\right]}^{-1/2}$ and ${Q}_{mc}={R}_{ms}^{-1}\sqrt{{M}_{ms}/{C}_{mc}}$ respectively).

The target parameters reads as

31 $${f}_{t}={f}_{0}\frac{{\mu }_{c}}{{\mu }_{m}},$$

32 $${Q}_{t}={Q}_{mc}\frac{\sqrt{{\mu }_{m}{\mu }_{c}}}{{\mu }_{r}},$$

where *μ*_*m*_ = *M*_*mt*_/*M*_*ms*_, *μ*_*c*_ = *C*_*mc*_/*C*_*st*_, and *μ*_*r*_ = *R*_*mt*_/*R*_*ms*_^84,90,96^.

A parametric study on individual tuning parameters by De Bono et al. revealed that the controller’s efficient bandwidth is predominantly influenced by the mass term *μ*_*m*_, although the compliance parameter *μ*_*m*_ also has a notable impact^92^. As an example, Rivet et al. achieved broadband absorption using the sensor/shunt based feedforward controller by synthesizing a matched impedance (*R*_*mt*_ = *ρc*) at 84 Hz with a bandwidth exceeding 410 Hz. This was accomplished by properly tuning the control parameters to *μ*_*m*_ = *μ*_*c*_ = 0.15 in this case^90^.

A key advantage of such a digital controller, based on a designed transfer function, is its flexibility in implementing a wide variety of target impedances. Notably, a multiple-degree-of-freedom (MDOF) target impedance can be synthesized, which is equivalent to a parallel connection of *n* single-degree-of-freedom impedances, i.e,

33 $${Z}_{st,n{\rm{DOF}}}(s)={\left[\mathop{\sum }\limits_{k = 1}^{n}{\left({Z}_{s{t}_{k}}(s)\right)}^{-1}\right]}^{-1}.$$

This MDOF target impedance extends the control bandwidth and enables assigning different target acoustic resistance values at discrete frequencies, providing a versatile framework for advanced applications^77^.

#### Narrowband control: complex envelope technic

In contrast, in some cases, the control must only be performed on a couple of specific frequencies over a narrow band. In such cases, fine-tuning the transfer function to get the appropriate behavior of the membrane may not be an easy task, and more adapted techniques exist such as the complex envelope technique^97,98^ which enables fine control over a given bandwidth *b*.

**Fig. 3:**
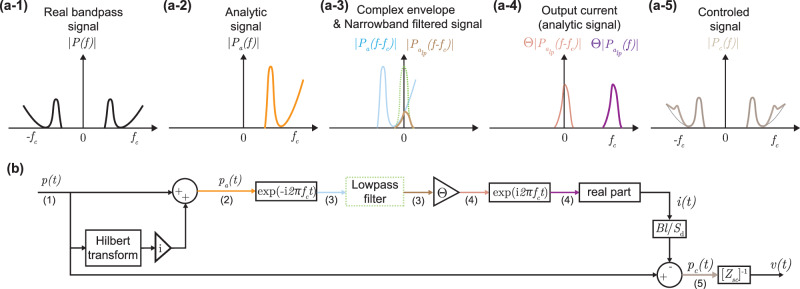
Complex envelope narrowband control strategy. **a** Illustration of the signal magnitude at the different steps of the complex envelope type control, **b** Block diagram of the control.

This signal processing technique allows applying a given transfer function to a band-limited signal. It involves several transformations, as described in the block diagram of Fig. 3b.

The analytical signal *p*_*a*_(*t*) of the sensed real pressure signal *p*(*t*) is first obtained by adding to it its Hilbert transform

34 $${p}_{a}(t)=p(t)+{\rm{i}}{\mathcal{H}}\{p(t)\}.$$

This first step has for consequence to suppress the negative frequency component of the real signal spectrum ∣*P*(*f*)∣, thus giving a single-sided positive spectrum ∣*P*_*a*_(*f*)∣

35 $${P}_{a}(f)=\left\{\begin{array}{ll}P(f)+{\rm{i}}(-{\rm{i}}P(f))=2P(f)\quad &{\rm{for}}\,f\, >\, 0\\ P(f)\quad &{\rm{for}}\,f=0\\ P(f)+{\rm{i}}({\rm{i}}P(f))=0\quad &{\rm{for}}\,f\, <\, 0.\end{array}\right.$$

Then, the analytic signal spectrum is shifted to DC by the frequency *f*_*c*_ at which the control has to be performed to get the complex envelope of the signal *p*_*e*_(*t*)

36 $${p}_{e}(t)={p}_{a}(t){{\rm{e}}}^{-{\rm{i}}2\pi {f}_{c}t}\Rightarrow {P}_{e}(f)={P}_{a}(f-{f}_{c}),$$

as illustrated by the light blue line in Fig. 3a-3

A low pass filter (e.g. second-order Bessel filter) is then applied to the complex envelope to limit the control on the target bandwidth Δ*ω* = 2*πb*, as illustrated by the dotted green and brown lines in Fig. 3a-3 respectively.

The control gain *Θ* is finally applied and the obtained analytic signal spectrum is shifted back to be centered at the control frequency,

37 $${i}_{a}(t)=\Theta {p}_{e}(t){{\rm{e}}}^{{\rm{i}}2\pi {f}_{c}t},$$

giving the analytical output current *i*_*a*_(*t*). The real output current *i*(*t*) to be injected into the loudspeaker is then simply obtained by taking the real part of the *i*_*a*_(*t*).

Such narrowband control has been used by Koutserimpas et al. to actively modify in a reconfigurable manner the loudspeaker impedance magnitude, resonance frequency, and quality factor, following the hybrid sensor-/shunt-based control^98^. A precise and accurate control was reported both on the closed-box loudspeaker and the free-field loudspeaker inside a waveguide. For the latter case, the block diagram of Fig. 2e is adapted by sensing both the front and rear pressures, and applying the control on the pressure difference.

### Sound absorption and isolation

#### Sound absorption with a single electrodynamic loudspeaker

**Fig. 4:**
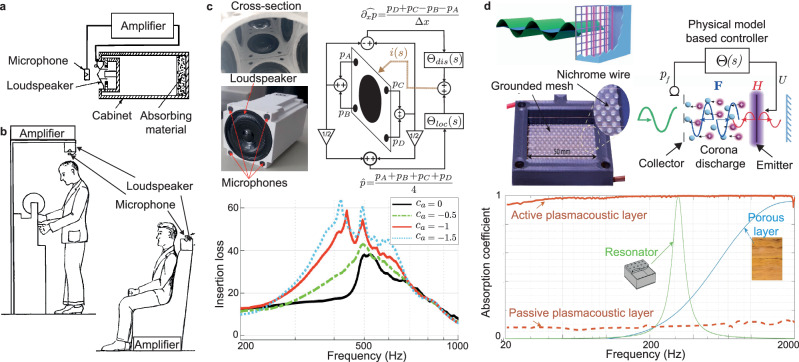
Active sound absorbers. **a** Original designs proposed by Olson and May, consisting of a loudspeaker fed by a direct impedance control law applied to sensed pressure in front of the closed-box loudspeaker with added absorbent layer adapted with permission from ref. ^99^ and **b** envisioned application of the design to create quiet zones in cars or offices adapted with permission from ref. ^99^. **c** Example of an active liner used to synthesize a nonlocal impedance along a channel, with and without airflow. Each unit cell comprises a controlled electrodynamic loudspeaker surrounded by 4 microphones to sense both pressure and velocity along the liner adapted with permission from ref. ^118^. The advection boundary control law (blue, red, and green lines) outperforms the local impedance control law (black line) as evidenced by the measured insertion loss for different convection speeds *c*_*a*_. **d** Schematic and photography of a plasmacoustic transducer, using the ionization of air between two electrodes to produce a sound wave with controlled particle and velocity (top panels). Both a monopolar heat source (*H*) and a dipolar force source (*F*) are generated when a sinusoidal signal is applied to the electrodes. Measured sound absorption in the 20–2000 Hz frequency range are compared with porous layer and resonators with equivalent thickness. A nearly perfect absorption is achieved over the whole frequency range thanks to the nonresonant behavior of the plasmacoustic metalayer(bottom panel). adapted with permission from ref. ^153^.

Building upon the foundational idea introduced by Olson and May^74,99^ (see Fig. 4a–b), impedance synthesis has been extensively developed to tune the reflection conditions at the interface of electrodynamic loudspeakers coupled with a velocity or pressure sensor in a feedback loop^60,78,81,100–103^. This method enables controllable and tunable acoustic absorption by actively adjusting the interface impedance.

In parallel, shunted loudspeakers combined with electronic circuits have been utilized to tune the resonant characteristics of loudspeakers, facilitating effective low-frequency and broadband sound absorption^75,86,104–106^. Hybrid designs incorporating passive treatments, such as porous materials or perforated plates, have also been proposed to complement actuators equipped with electronic shunts^107–110^ or active feedback control systems^102,111,112^. Although these hybrid systems extend the effective absorption bandwidth, their adaptability, and tunability remain inherently limited.

As reviewed in “Different control scheme”, various active control strategies—ranging from direct impedance control to feedforward techniques and advanced controllers designed to minimize the effects of uncertainties in loudspeaker characterization—have been developed primarily for acoustic absorption. By enabling tunable impedance matching, these approaches have driven significant advancements in the field of space-constrained acoustic absorption, addressing the challenges of achieving high-performance sound control within compact environments.

#### Sound absorption with arrays of transducers and hybrid active/passive structures

Practical implementations of active sound absorption devices often involve building distributed source liners along the walls of ducts or nacelles to control sound transmission and propagation in multiple directions^113–122^. For liners addressing one (resp. two) dimensional wave propagation, a unit cell typically consists of a loudspeaker, at least 2 microphones, i.e., one upstream and one downstream of the loudspeaker (resp. 4 microphones placed around it), and a dedicated controller as depicted in Fig. 4c. Two main strategies are employed to achieve absorption with such configurations: locally reacting and non-locally reacting liners. The impedance of these liners is generally expressed as

38 $${Z}_{t}\frac{\partial {v}_{n}}{\partial t}=\frac{p}{\partial t}-{c}_{a}\frac{\partial p}{\partial x},$$

where *v*_*n*_ is the parietal normal velocity and *c*_*a*_ is the transport speed of the advection condition^114^. If *c*_*a*_ = 0, the impedance corresponds to a locally reacting liner.

For non-local implementations, an additional term involving spatial differentiation of the pressure measured between two consecutive sets of microphones needs to be accounted for. In refs. ^123,124^, Collet et al. developed an experimentally validated theoretical framework for achieving absorption within ducts using one- or two-dimensional arrays of loudspeakers mounted along the walls of a waveguide. Each loudspeaker is independently controlled using partial differential equation (PDE) control theory. This non-local strategy enables targeting an impedance that depends on both frequency and wavenumber, i.e., *Z*_*st*_ = *Z*_*st*_(i*ω*, i*k*). By imposing a local skin velocity *v*(*x*, *y*, *t*) as a function of the pressure measured along the grating, co-localized controllers enhance both the efficiency and versatility of the control, increasing the degrees of freedom to process incident waves and annihilate reflections^123,124^. This voltage-based approach has been extended to current-driven liners^114,125^. In such systems, the control current is defined as

39 $$i(s)={\Theta }_{loc}(s)p(s)+{\Theta }_{dis}(s)\frac{\partial p}{\partial x}$$

where

40 $${\Theta }_{loc}(s)=\frac{1}{Bl}\left({S}_{d}-\frac{{Z}_{ms}(s)}{{Z}_{mt}(s)}\right){\rm{and}}\,{\Theta }_{dis}(s)=\frac{{c}_{a}{Z}_{ms}(s)}{sBl{Z}_{dis}(s)},$$

are the local and distributed control laws respectively. For absorption application, the local target impedance can be set as *Z*_*loc*_ = *ρc*, while the distributed target impedance can be defined as *Z*_*dis*_ = i*ωρ*.

In contrast, locally reacting liners can be directly controlled using the mean pressure measured by the 4 microphones. The classical control laws described in “Different control scheme” can be applied to achieve broadband noise reduction, even under grazing flow conditions^113^. Billon et al. characterized both locally and non-locally reacting 2D liners, with and without flow (up to Mach 0.29). Their study demonstrated strong absorption performance and control stability within the 300–1500 Hz frequency range. Notably, implementing the non-local strategy improved absorption by 0.1 and increased insertion loss by 3 dB. Figure 4d shows that the advection boundary control law (blue, red, and green lines) outperforms the local impedance control law (black line) in terms of insertion loss, for the different convection speeds *c*_*a*_ measured^118^.

Alongside fully active liners, hybrid passive/active metamaterials have been developed to enhance the performance of passive acoustic metamaterials. Systems such as Helmholtz resonators^126–129^ or quarter-wavelength waveguides^130–132^ augmented with actively controlled loudspeakers have demonstrated superior performance compared to the passive resonator. Cheer et al. further showed that such hybrid active/passive metamaterials can outperform the performance of purely active control^132^.

Inspired by the membrane and plate-type metamaterial with added static^19,133^ and statically actuated^37,38^ platelets, Langlfeldt et al. proposed the integration of an active electrodynamic actuator coupled to a sensor within the membrane of the unit cell. This design allows real-time reconfiguration of its acoustic properties through direct control of the measured frontal pressure, enabling on-demand shifting of the anti-resonance frequency at which the membrane ceases to transmit sound^134^. Their results demonstrated that a simple proportional gain controller could shift the anti-resonance frequency over an octave, achieving a sound transmission loss greater than 15 dB with minimal input voltage. In ref. ^135^, the authors explored different feedback control schemes for their active metamaterial, namely applying a feedback control combining the sensing of the pressure inside the cavity, the mass acceleration, and the mass displacement. They reported that this straightforward feedback loop with constant gain enables broad, deep-subwavelength frequency range reconfiguration in a compact design. The absorption peak, exceeding 85%, could be adjusted at will across various ranges: 285–690 Hz with cavity pressure feedback, 265–380 Hz with mass acceleration feedback, and 285–550 Hz with mass displacement feedback^135^.

In the same spirit, Wu et al. reported a programmable shunted electromechanical diaphragm having a broadband and deeply subwavelength insertion loss exceeding 20 dB over 5.7 octaves (from 15 to 772 Hz)^136^. Shunted piezoelectric actuators have also been employed to enhance the performance of passive acoustic metamaterials, further broadening their absorptive capabilities^137–139^.

#### Nonlinear active control for improved absorption

The concept of energy pumping using a nonlinear energy sink, extensively studied in mechanics^140^, has also been successfully applied to acoustics through nonlinear membranes^141–143^. The underlying idea is to use a nonlinear resonator to extract and dissipate energy from a linear system. Enhanced versions of passive energy sinks have been demonstrated using hybrid systems, such as cubic nonlinear membranes coupled with either an electrodynamic loudspeaker under proportional feedback control^144^ or a loudspeaker connected to a passive nonlinear shunt^145^.

Inspired by these works, Guo et al. proposed a novel strategy to control the nonlinearity of a closed-box loudspeaker at low intensities. Their method combined active linear feedforward control based on measured front pressure with additional nonlinear feedback control derived from the diaphragm velocity, estimated using the rear pressure. This innovative approach significantly enhanced the absorption performance in terms of both bandwidth and absorption values^146^. Specifically, the introduction of a synthesized cubic nonlinearity allowed the efficient absorption bandwidth (defined as *α* > 0.8) to double compared to that achieved with linear control while transferring only 0.13% of the incident energy into higher harmonics. De Bono extended this idea by proposing a general framework for achieving locally responsive causal dynamics, including time-varying and nonlinear targets, through a pressure-based current-driven architecture, similar to hybrid/shunt sensor-based impedance control, but with a model inversion scheme based on real-time integration^147^. The framework was demonstrated with a Duffing oscillator as the target dynamics. Morell et al. further generalized this methodology to encompass a wide range of nonlinearities, including cubic, stepwise linear, polynomial, and logarithmic nonlinearities^148^. Their work underscores the potential of such nonlinear control strategies for active noise attenuation, even with low excitation amplitudes, opening new avenues for advanced sound absorption technologies.

#### Sound absorption with actively controlled plasmacoustic transducers

Active sound absorbers are mostly based either on piezoelectric membranes and plates or electrodynamic loudspeakers, which are easy to implement and have a relatively wide frequency range suitable for many applications. They are also inexpensive and easy to characterize and model analytically. However, their behavior relies on mechanical parts that can degrade over time, introduce unwanted non-linearities and, most importantly, resonate, limiting the working bandwidth around their resonant frequency, and being mechanically opaque thus scattering sound waves. As a result, many applications require multiple loudspeakers of different bandwidths to cover the entire audible frequency range thus increasing the complexity and weight of the overall structure.

A new type of transducer, the plasmacoustic or plasma-based actuator, already used in the literature for flow and instability jet control^149,150^, has recently been proposed for active sound absorption^151–153^. These actuators consist of two metallic electrode grids separated by a dielectric gap, as shown in the schematic in Fig. 4d. When a high-voltage dc electrical voltage is applied to the transducer, a constant ionization of the air occurs. Combining an alternating high-voltage signal results in a controllable volume force that can be used to impart a controlled particle velocity around the actuator and thus perturb the local pressure field and generate controlled sound. The major advantage of such transducers is the absence of moving parts in their design, which makes them robust, lightweight, almost transparent to sound waves, and non-resonant.

Using two microphones in front of the actuator to estimate the local particle velocity of the closed box actuator, Sergeev et al. used a pressure-velocity feedback loop for direct impedance control. Both a hybrid passive/active configuration with additional wire mesh between the microphone pair and an additional layer of melamine in the cabinet, and a purely active configuration were tested^152^ in both normal and grazing incidence. Broadband sound absorption (under normal incidence) and transmission loss (under grazing incidence) were reported in the range 100 Hz–2000 Hz. The authors later reported the high performance of their transducer to control arbitrary reflections and also an almost perfect absorption down to 20 Hz^153^. It is worth noting that, in theory, there are no strict lower limit, the low frequency range being only determined by the measurement capabilities at low frequencies, while the higher frequency limit is fixed by the chosen controller.

### Room acoustics correction

Building on the exceptional performance of single or multiple controlled transducers, active control strategies have been developed to fine-tune room acoustics, balancing reverberation levels and intelligibility. Active reverberation enhancement systems have been a primary focus, using distributed loudspeakers in enclosed spaces to process and retransmit sound measured from different locations, and allowing control of reverberation and sound amplification^154–157^. Three main approaches have been proposed and commercialized: in-line systems, regenerative (or non-in-line) systems, and hybrid systems. In in-line systems, directional microphones placed near sound sources (e.g., on stage) capture audio signals, which are digitally processed and transmitted through loudspeakers distributed around the room. These systems recreate artificial early reflections and reverberations, improving sound quality and spatial distribution^158–162^. Regenerative systems, in contrast, use distributed microphones and loudspeakers to directly feed back detected pressure to the loudspeakers with specified gain, delay, and digital processing, which can include added reverberation^163–166^. These strategies have been commercialized and successfully implemented in numerous concert halls and performance venues, improving acoustics for diverse settings^167,168^.

Expanding on regenerative approaches, active walls have been developed to modify and control acoustic properties in a space. Early work by Guicking et al. in 1985 introduced the concept of active walls using a 3 × 3 loudspeaker array^80^. By using microphones to measure the front pressure and applying a feedback loop with adjustable electronics (phase shifter and controllable gain), the authors achieved arbitrary control of reflection at both normal and oblique incidence, setting the stage for active control for room mode absorption and controllable diffraction. A few years later, the active wall concept was improved with the introduction of adaptive filters^169,170^ and feedback controllers^82,162,171^.

In 2022, Gao et al. proposed a similar active wall, consisting of piston-like actuators actively moved by a piezoelectric element, and capable of achieving a range of boundary conditions from soft to hard, thus enabling reflection and absorption in a room to be controlled^172^. A demonstration of active multiple-input multiple-output communication channels in disordered environments, replicating the cocktail-party effect, was also reported using convolution filters and time-reversal strategies in the ultrasonic regime^173^.

Active control schemes can also be used to control the low-frequency resonant modes of a room that cause uneven distributions in space and frequency and can alter the quality of the temporal acoustic response, e.g. flatter echos, abnormal strong resonances or antiresonances. As shown in ref. ^113^, control schemes can be used to damp mode in a duct, and can be extended to room modal equalization^77,174–176^.

Finally, dedicated quiet zones can be achieved in rooms and enclosures by using one or several secondary sources under feedforward or feedback control laws^177–181^ or in contrast, personalized sound zones^182–184^.

## Application of active metamaterials: global control of a structure’s effective properties in space and time

Hereafter, we shift our focus from using active control to modify the properties of individual transducers to its application in active metamaterials, i.e., the use of active control techniques to engineer and manipulate the effective properties of entire structures or metamaterials. Specifically, we will review recent advancements such as active modulation in space and time, gain and loss control, and non-reciprocal coupling.

### Control of the dispersion and effective parameters

As previously discussed, the exotic behaviors achievable with passive metamaterials are often constrained by severe limitations in bandwidth, governed by the Kramers-Kronig relations, and by their lack of reconfigurability. Active control techniques overcome these limitations, enabling the adjustment of a material’s effective parameters over a wide bandwidth and range of values^72,73,185–192^. The arbitrary control of the effective properties of metamaterials made of piezodiaphragms has been the focus of extensive work (both analytical and experimental) carried out by Akl and Baz^71,72,193–196^. Popa et al. also highlighted the possibility of dynamically and independently tuning the effective mass density and bulk modulus of a system, even to negative values and negative refraction^185^. To do this, they used a purely electronic feedback control loop between a sensing transducer (unidirectional electret transducer) and a driving transducer (piezoelectric diaphragm or monopole-like counterfacing transducers). Interested readers are referred to the following detailed review of piezoelectric acoustic metamaterials for an overview of the specific use of piezoelectric elements^139^.

**Fig. 5:**
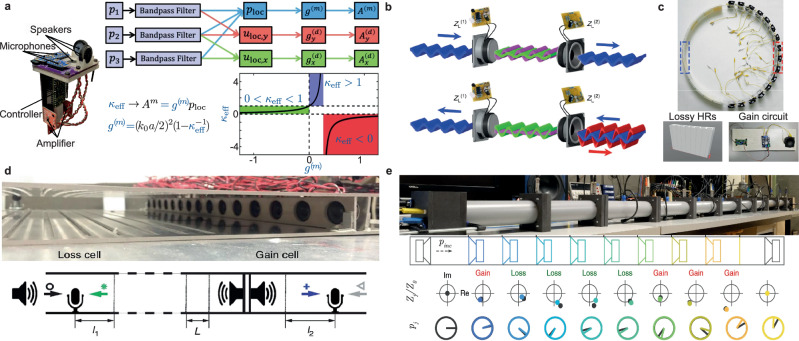
Effective parameters and non-Hermitian acoustics. **a** Unit cell composed of three orthogonal loudspeakers and microphone allowing to estimate the local pressure and velocity in the xy plane and control both monopolar and dipolar modes. This fine control allows to engineer independently the effective bulk modulus and dynamic mass density by applying constant gain feedback on *p*, *v*_*x*_, and *v*_*y*_ adapted with permission from ref. ^186^. **b** PT-symmetric system consisting of two loudspeakers loaded with non-Foster electrical circuits, by means of which the gain and loss can be compensated, enabling non-intrusive sensing adapted with permission from ref. ^209^. PT-symmetric hybrid passive/active system composed of lossy Helmholtz resonators adapted with permission from ref. ^210^ **c** or side-branched slits adapted with permission from ref. ^203^ **d**, the viscothermal losses of which is compensated by active electrodynamic loudspeakers. **e** Non-Hermitian acoustic metamaterial evidencing constant pressure propagation thanks to fine control of losses and gain synthesized by 10 actively controlled loudspeakers (sensor/shunt control). The target impedance and necessary gain and loss to be synthesized along the propagation path are shown at the bottom of the photography adapted with permission from ref. ^213^.

More generally, virtualized, i.e. actively controlled meta-atoms composed of loudspeakers and microphones, can be used to adapt a system’s dispersion relation at will^190,191,197,198^, and thus realize transformative acoustics^198,199^, active cloaking^199–205^ and broadband impedance matching^206^. In particular, Kovacevich et al. have designed a unit cell capable of independently controlling the monopolar (proportional to pressure) and dipolar (proportional to local particle velocity) response of the metamaterial^186,191,192^, using simple gain and phase-shift feedback loops combining pressure measured by three different microphones and feeding three orthogonal loudspeakers assembled on a chip shown in Fig. 5a. This design therefore allows an independent control of the bulk modulus and effective dynamic mass density. The former can be controlled by directly applying a constant gain to the local pressure, while an anisotropic mass density tensor can be designed by applying different gains on the particle velocity in *x* and *y* direction in the feedback controllers that drive two of the orthogonal speakers. By adequately choosing the mass density and bulk modulus, one can then achieve full control of the dispersion, and therefore any acoustics properties, e.g. impedance matching or slow sound among others.

### Control of gain and loss: non-Hermitian acoustics

While passive acoustic metamaterials and resonators inevitably suffer from detrimental visco-thermal losses^207^, active cells can compensate for dissipation. In particular, systems simultaneously respecting parity (P) and time (T) symmetries provide a perfect platform for loss-immune acoustic metamaterials, as in this regime, losses are perfectly compensated by gain opening the path to unidirectional cloaking, also known as anisotropic transmission resonance (ATR). Although PT symmetry can be achieved passively, e.g. by properly coupling designed diaphragms interacting with flows^208^, active control provides more tunability and freedom. In ref. ^209^, Fleury et al. used two loudspeakers shunted by non-Foster electronics, allowing to tune gain and loss in the system appropriately and achieve unidirectional invisibility, thus realizing a loss-immune sensor (see Fig. 5a). Hybrid passive designs have also been reported, using lossy resonators (slits or Helmholtz resonators) for the loss cells and arrays of controlled loudspeakers for the gain part^203,210^ as shown in Fig. 5d, e respectively. The distance between the gain and loss cells can also be optimized to tune the PT-symmetric system^210^.

In addition, engineering losses and gains in a complex disordered system can mitigate the complex propagation challenge posed by multiple scattering and enable constant unitary transmission along disordered medium^211,212^. The experimental demonstration of these theoretical works has been reported on a discrete acoustic system by Rivet et al.^213^. Using an array of actively controlled non-Hermitian electrodynamic loudspeakers located inside the disordered waveguide depicted in Fig. 5e, the authors were able to control the specific impedance of each resonator using the hybrid sensor-shunt technique to add the appropriate gain and loss distribution, enabling constant-pressure propagation. In this way, the opaque disordered medium becomes completely transparent when actively controlled.

### Control of phased-arrays for reconfigurable steering, multiplexing, and lensing

Since the impedance of actively controlled resonant cells can be tuned in both amplitude and phase, and negative effective parameters can be achieved, these structures can be used to spatially manipulate waves, enabling reconfigurable steering and focusing^214–218^, as well as abnormal refraction, among other things.

In ref. ^215^, Popa et al. demonstrated the ability of a layer of 10 actively controlled piezoelectric membranes to focus three successive incident plane wave pulses at three different locations, reproducing a lens, thanks to dedicated reconfigurable electronics based on a feedforward loop. They were also able, with the same setup, to steer the waves in two different directions simultaneously, thus reproducing a multiplexer. Membrane-type metamaterials with variable tension, tunable by piezoelectric transducers or an external magnetic field, have been used to focus and realize flat lenses, self-bending beams, and cloaking surfaces^216–218^.

In ref. ^219^, Lissek et al. extended the concept of diffraction pattern control by membrane-type metamaterials to an array of active electroacoustic resonators. Applying the sensor/shunt-based control scheme, the authors demonstrated the possibility of adequately controlling the reflection phase and amplitude of each of the unit cells composed of an electrodynamic loudspeaker to steer the incident acoustic wave in a given direction or with a given reflection directivity over a wide frequency bandwidth. Zhai et al. then reported in 2021 an experimental realization of a 3 × 3 omnidirectional active metasurface capable of controlling the diffraction pattern in free space^220^.

By precisely controlling the independent phase of each transducer in a parametric phased array, it is possible to produce specific wave beams. These beams can be used to focus energy, steer acoustic waves in a desired direction, or perform advanced tasks such as wavefront shaping and holography. Although this does not stem directly from an active control scheme based on pressure or velocity sensing (as discussed in “Electroacoustic transducer modeling”), such control might still be classified as active. Specifically, space-time phase modulation enables sophisticated manipulation of acoustic waves, including frequency-selective beaming in both transmission and reception (detection) modes^221^ and precise diffraction pattern control^222^. Furthermore, combining passive metamaterials with dynamic phased arrays has been shown to enhance sensing and communication performance while reducing the number of required transducers^223,224^.

### Space and/or time modulated structures

**Fig. 6:**
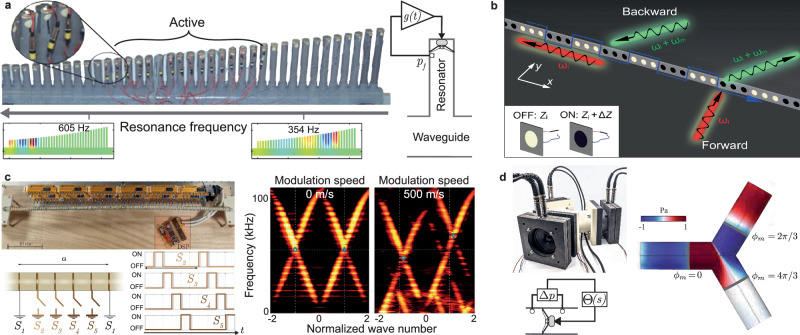
Spatial and temporal applications. **a** Gradient index metamaterials composed of quarter wavelength detuned resonators augmented by an actively controlled loudspeaker under feedback amplification, reproducing the tonotopic frequency mapping of the cochlea with hypersensitivity to low amplitude sounds adapted with permission from ref. ^229^. **b** Metasurface of shunted parallel loudspeakers with time-varying impedance leading to mode transition and non-reciprocal propagation at different Floquet harmonics adapted with permission from ref. ^233^. **c** Linear array of 113 piezoelectric elements under time-varying grounding conditions that allows the measurement of the complete band diagram of the space-time metamaterial adapted with permission from ref. ^236^. **d** Airborne acoustic circulator based on spatiotemporal modulation of the synthesized moving mass of three loudspeakers terminating a three-port network adapted with permission from ref. ^239^.

As shown in the previous sections, active cell arrays have demonstrated their ability to manipulate acoustic waves in reflection and transmission, and to achieve exotic effective parameters and properties. Local real-time feedback control of an array of resonant transducers can also be used to implement gradient-index locally resonant materials to control the spatio-frequential propagation of an acoustic wave on the principle of rainbow trapping systems^225–227^ and mimic complex continuous systems such as the cochlea^228–230^. Rupin et al. used a liner of tuned quarter-wavelength resonators terminated by electrodynamic loudspeakers, illustrated in Fig. 6a. While the passive system reproduces well the cochlear tonotopic mapping of frequency as a function of position along the system, the active feedback reproduces the hypersensitivity to low amplitude signals. In this case, the active resonators operate close to a Hopf bifurcation, tuning the response as a function of signal amplitude^229,230^.

The integration of time modulation together with spatial modulation has opened new avenues for wave manipulation as reported in these recent reviews^231,232^, although experimental implementations are still technically challenging and the achievable modulation frequency *f*_*m*_ = *ω*_*m*_/2*π* remains slow most of the time. The versatility of actively controlled systems allows these challenges to be addressed. Time-varying resonant behavior can be obtained either by alternately turning on and off the shunt of a loudspeaker or a piezoelectric transducer (see Fig. 6b)^233–236^ or by modulating periodically in time and at any frequency the active control law^189,237–239^, leading to frequency conversion and Floquet harmonic generation^233,237,238^, mode and topological phase transition^233,240^, wave steering and focusing^233^, unidirectional amplification^241^, and non-reciprocal propagation^237,239,241,242^. In ref. ^236^, Tessier-Brocéliande et al., experimentally demonstrated and measured the full dispersion diagram of a periodic array of 113 piezoelectric elements with periodic switching of their ground state (see Fig. 6c).

Loudspeakers with time-varying mechanical parameters can also be synthesized by adapting the control law *Θ*(*t*) of the hybrid Sensor-/Shunt controller as^238^

41 $$\Theta (t)=\frac{Sd}{Bl}\left(1-\frac{{Z}_{sc}}{{Z}_{st}(t)}\right),$$

where the target-specific impedance, now varies with time and can be designed at will. The classical modulation function read as follows ${Z}_{st}(t)={Z}_{st}(s)\left(1+{A}_{m}\cos \left({\omega }_{m}t+{\phi }_{m}\right)\right)$ or ${Z}_{st}(t)=s{\mu }_{m}(t){M}_{ms}/{S}_{d}+{\mu }_{r}(t){R}_{ms}/{S}_{d}+{\mu }_{c}(t){(s{C}_{ms}{S}_{d})}^{-1}$, with time modulated coefficient $\mu (t)=\mu \left(1+{A}_{m}\cos \left({\omega }_{m}t+{\phi }_{m}\right)\right)$.

Due to the modulation, Floquet harmonics are generated around the excitation frequency, at multiple integers of the modulation frequency *ω*_*n*_ = *ω* ± *nω*_*m*_, where *n* is the number of harmonics generated. There is therefore a transfer of energy that can be used to attenuate certain frequencies by transferring them to infrasound, outside the audible range, and therefore less affected by obstacles along the propagation path^243^. An asymmetric transfer and a transformation from monotonic to white noise can be achieved by replacing the classical cosine modulation with a complex exponential^238^ or a random modulation^243^ respectively.

Time modulation also allows breaking the time-reversal symmetry without a magnetic field. In ref. ^237^, three coupled cavities equipped with microphones and loudspeakers were used to demonstrate synthetic magnetism and frequency conversion. By modulating the coupling strength between the cavities in time, large non-reciprocal propagation with tunable high isolation was observed. Time-varying feedforward active control applied to three loudspeakers placed at the exit of a “Y”-shaped triport network was also used to synthesize an effective momentum bias in the system, mimicking the Zeeman effect in quantum mechanics^239^. A cosine modulation of the moving mass, i.e. ${\mu }_{m}(t)={\mu }_{m}\left(1+{A}_{m}\cos ({\omega }_{m}t+{\phi }_{m})\right)$, with *ϕ*_*m*_ = 0; 2*π*/3; or 4*π*/3, was synthetically applied to each of the loudspeakers to demonstrate the audible airborne sound circulation in the system shown in Fig. 6d.

### Asymmetric and non-reciprocal propagation

**Fig. 7:**
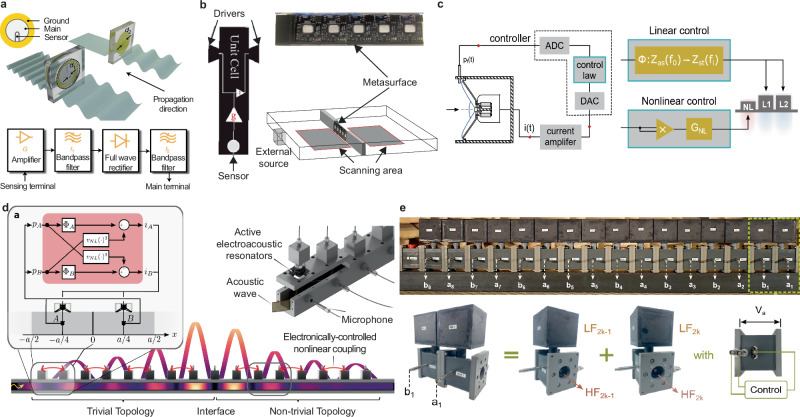
Nonreciprocal and topological transport applications. **a** piezoelectric membrane under feedback control sandwiched between two asymmetric Helmholtz resonators offering impedance matching, used to evidence strong isolation and nonlinearity adapted with permission from ref. ^247^. **b** 5 unit cells metasurface composed of counter facing transducers and microphones allowing to reproduce monopolar and dipolar modes and to engineer a broadband sound barrier adapted with permission from ref. ^255^. **c** liner of three controlled loudspeakers used to evidence a nonreciprocal frequency conversion of real sound. The first control synthesizes nonlinearity aiming to to generate the harmonic of the incident signal, while the two other linear controlled speakers aim at canceling the fundamental signal adapted with permission from ref. ^258^. **d** Liner of 16 controlled loudspeakers introducing nonlinear and nonlocal coupling in a waveguide. By engineering the coupling type between each unit cell, an interface state can be observed, for which a topologically protected confinement of sound is observed adapted with permission from ref. ^263^. **e** Hyrbid metamaterials composed of an array of Helmholtz resonators coupled by actively controlled loudspeakers evidencing a zero-energy edge state adapted with permission from ref. ^265^.

Breaking the fundamental reciprocity property of waves, i.e. the ability to swap emitters and receivers without changing the acoustic response, enabled the development of applications with unprecedented propagation behavior, e.g. diode, isolator, gyrator, etc. Although easily achieved in electromagnetics by using ferromagnetic materials, breaking reciprocity remains a challenge in acoustics at low power levels due to the low magneto-acoustics effect on sound waves. Since both nonlinearity and time modulation can be achieved by active control, strategies can be developed to exploit nonreciprocity in acoustics^244^. Broadband nonreciprocal acoustic scattering has been reported, for example, using a loudspeaker with asymmetric feedback^245^, loudspeaker pairs driven by an adaptive filter^246^, active piezoelectric membrane sandwiched between two asymmetric Helmholtz resonators as shown in Fig. 7a^185,247^, an active line array with programmable boundary conditions^248^, or local and nonlocal active liners^249–251^. With these designs, unidirectional amplification^241^, absorption or isolation^247,249^, diode^252^ or gyroscopic nonreciprocity^253,254^ were observed.

A particular group of asymmetric and nonreciprocal materials has attracted considerable attention, the Willis materials, that present a coupling between potential and kinetic energy in their constitutive equations. The use of actively controlled loudspeakers has allowed demonstrating tunable and strong Willis coupling in active linear Willis medium^187,194,197,241,252,255,256^. In particular, Popa et al. demonstrated the potential of bianisotropic (Willis) active metasurface as an efficient broadband sound barrier. The unit cell is designed with two back-to-back loudspeakers and a microphone, as shown in Fig. 7b so that both monopolar and dipolar response can be controlled in the feedback gain^257^.

Finally, Guo et al. reported a nonlinear and non-Hermitian active liner capable of unidirectional frequency harmonic conversion in real sound^258^. By ingeniously combining three loudspeakers, one under nonlinear feedback control, that generates the desired harmonic content, and two under liner hybrid sensor-/shunt linear controllers to block the fundamental frequency. The nonlinear feedback controller applies a constant gain *G*_*NL*_ and elevates the sensed pressure to a given nonlinear power law *α*_*NL*_, i.e., $i(t)={G}_{NL}| {p}_{f}{| }^{{\alpha }_{NL}}$. Similar nonlinear control has also been used to artificially increase nonlinearities in multimode cavities used for neuromorphic computing^259,260^.

### Condensed matter physics analogs and topological transport

The versatility of active metamaterials also makes them a valuable platform for mimicking and reproducing condensed matter physics and photonic behavior at the macroscopic scale. Topologically protected interface states, transport, and insulators are some of the stringent examples of achievable applications.

Acoustic analogs of the Su-Schrieffer-Heeger (SSH) system^261^, i.e., a 1D lattice of coupled dimers hosting interface state, and of the Hatano-Nelson model, i.e, a one-dimensional (1D) lattice with asymmetric hoppings responsible for bulk edge states and non-Hermitian skin effect^262^, have been demonstrated using controlled electrodynamic loudspeakers with sensor/shunt based control and feedback control respectively.

Active control schemes have the interest on top of tunable coupling and impedance synthesis, to generate controlled nonlinearities. Padlewski et al, demonstrated a robust amplitude-driven topological confinement of sound^263^ with a liner of 16 controlled speakers used to engineer the nonlinear couplings between sites. Each unit cell of the metamaterial consists of two closed-box loudspeakers placed on the wall of a rectangular waveguide. The controlled currents driving each speaker *A* and *B* are composed of a local impedance synthesis part (hybrid sensor/shunt method used to reduce the losses in the system), and a nonlocal nonlinear coupling, where the pressure sensed in front of speaker *A* is used to drive speaker *B* after the nonlinear control law. In the first half of the metamaterial, the control law is set so that an intra-cell coupling is favored, while an intercell coupling is synthesized in the second part, giving rise to the topologically protected confinement at the interface. With a similar set-up, the authors also demonstrated amplitude-driven energy guiding^264^.

With a hybrid design combining passive Helmholtz resonators coupled through waveguides with embedded active loudspeakers, Guo et al. reported a zero-energy edge-state with strong topological protection using chiral nonlinearities synthesized by the loudspeakers coupling two adjacent Helmholtz resonators^265^ as illustrated in Fig. 7e.

Time modulation can also be used to tailor topological phases and design Floquet topological insulators^53,266^. In ref. ^240^, Chen et al. designed a 1D acoustic lattice of 10 air-filled cavities dynamically coupled by loudspeakers driven by a constant gain feedback loop modulated periodically by double-pole, double-throw (DPDT) relays (a similar design was also proposed in ref. ^267^). As a result, the designed time-varying Su-Schrieffer-Heeger system allowed the observation of nontrivial Floquet *π* modes.

These works show the high potential and flexibility of active sound control techniques and pave the way for their use in even more complex condensed matter physics (e.g. higher dimension topology).

## Conclusions and perspectives

Active noise mitigation has a long and rich history, with significant advances driven by the need for improved acoustic environments and noise control solutions. The commercialization of room acoustic enhancement systems for multi-purpose venues and the implementation of Active Noise Cancellation (ANC) in everyday commercial devices, such as headsets, demonstrate the practical impact of this field of research. Despite the maturity of ANC techniques, ongoing research continues to refine algorithms, expand operational frequency ranges, and improve robustness and quality, especially in response to complex and external random noise events^67,268–270^. In addition, the adoption of ANC in industries such as aviation, rail, and automotive underscores its importance in improving user comfort and mitigating environmental noise, a pressing concern due to its well-documented adverse effects on human health. Efforts to address noise pollution have also extended to innovative applications of ANC, such as noise reduction windows^271,272^ and barriers^273^.

In parallel with the research on noise cancellation techniques, controlled sound absorption, focusing, and diffusion using passive and/or active strategies have also received considerable attention. While passive solutions offer simplicity, cost-effectiveness, and independence from external power sources, their inherent limitations - frequency bandwidth, frequency-dependent size constraints, and lack of adaptability - make active approaches a compelling alternative. In particular, active impedance synthesis control strategies enable unprecedented reconfigurability while maintaining compact designs, although they require advanced electronics and external power infrastructure. However, it is worth noting that the electronics can be centralized and detached from the treatment, which simplifies their integration in practical settings. We have compared and summarized in Table 2 the main differences and characteristics of both passive and active design strategies.

**Table 2:** Comparison and characteristics of passive and active acoustic treatments in a nutshell

Characteristics	Porous materials	Static passive metamaterials	Mechanically tunable metamaterials	Activelly controlled transducers
Principle	Interconnected dissipative pores	Resonant based	Tunable resonance-based	Actively tunable resonators
Frequency range	- Broadband, - Medium/high frequency	- Narrow band, - Increased bandwidth with collective resonances	- Narrow band, - Increased bandwidth with collective resonances	- Narrow-band for resonant transducers - Broadband for plasma transducers, - Broadband impedances can be artificially synthesized
Reconfigurability	- Static, - Not reconfigurable, - One application per design	- Static, - Not reconfigurable, - One application per design	- Mechanically reconfigurable (limited by design), - Several applications possible	- Electronically reconfigurable at will
Dimension	- Quarter wavelength, - Frequency dependent	Subwavelength	Subwavelength	- Intrinsically subwavelength for the transducers themselves - But requires additional electronics and power suppliers
Weight	- Lightweight (foams, wool,..), - Size and frequency dependent, - e.g. *ρ* ≈ 1.6 g.cm^−3^ (melamine).	- Light/medium weight: mainly air-filled 3D printed cavities/resonators or thin membranes, - e.g. *ρ* ≈ 1 g.cm^−3^ (filament)	- Light/medium weight: mainly air-filled 3D printed cavities/resonators or thin membranes + weight added mechanism	- Lightweight for piezo diaphragms, - Lightweight, nonresonant & transparent for plasma transducers - Medium weight for loudspeakers, + Medium/high for the required external electronics.
Real world integration	- Easy, - Widely implemented for room acoustics and sound absorption, - Limited cost - Durability	- Still in development and industrialization phase (mostly for noise mitigation) - Limited cost thank to 3D printing	- Still at the research and development phase - Medium cost and integrability due to the added mechanics	- Still at the research and development phase for sound absorption and active metamaterial purposes (possible application for sensing, routing, communication,…) - Widely implemented and commercialized for ANC (headsets, …) and room acoustics enhancing systems - Medium cost and integrability due to the added electronics - Transducer dependent durability

Although primarily driven by noise reduction and room acoustic treatment, the versatility and flexibility of active control and impedance synthesis also unlock new experimental capabilities to explore wave physics. Active systems provide a platform for investigating phenomena such as non-Hermitian acoustics, non-reciprocal wave propagation, and topological behaviors. For example, actively controlled loudspeakers or piezodiaphragms provide a simple experimental platform for exploring space-time modulated media and analogues of condensed matter phenomena. The prospect of actively controlled plasma and other non-resonant transducers is also a promising research direction in this rapidly developing field, as they are inherently broadband and transparent, and can therefore be easily integrated into ventilated systems.

This review has explored the wide range of active control strategies. From the simplest proportional controller to more advanced schemes such as mixed feedforward/feedback, the vast zoology of active control schemes makes it possible to find the most appropriate strategy for each situation, depending on the precision and stability required by the application being pursued. While proportional and feedback control remain foundational approaches, achieving precise impedance synthesis often requires advanced techniques and detailed transducer modeling, as well as a careful input/output latency management.

Beyond acoustics, similar control schemes are being used to manipulate elastic waves, enabling efficient vibration damping^274^, effective properties engineering^275^, and exploration of space-time modulated^276,277^ and non-Hermitian physics^278,279^ and non-reciprocal phenomena^280^ or topological interface states^281^ in solid mechanics.

In closing, the field of active noise mitigation and wave control is poised for transformative advances. The versatility and potential of active control systems offer not only practical sound manipulation capabilities, but also avenues for groundbreaking scientific exploration. Further developments in hybrid active-passive strategies hold great promise for bridging the gap between current capabilities and real-world applications and could improve diverse technological domains including but not limited to adaptive room acoustic and noise mitigation, hearing aids devices and implants, personal sound zones and quiet zones, automotive, space and aeronautic industries, telecommunication, sensing, or routing. We envision a future rich in innovative solutions that address both current challenges and new dimensions in wave physics and materials science.
